# Cardioprotective strategies of ACEi/ARBs and beta-blockers against anthracycline-induced cardiotoxicity in pediatric cancer survivors: a systematic review

**DOI:** 10.1186/s40959-026-00447-5

**Published:** 2026-02-18

**Authors:** Reza Sattarpour, Maryam Noori

**Affiliations:** 1 Women’s Reproductive Health Research Center, Tabriz University of Medical Sciences, Tabriz, Iran; 2 BreastFeeding Research Center, Family Health Institute, Imam Khomeini Hospital Complex, Tehran University of Medical Sciences, Tehran, Iran; 3 Center for Health Related Social and Behavioral Sciences Research, Shahroud University of Medical Sciences, Hafte Tir Square, Shahroud, 3614773955 Iran; 4 Network of Immunity in Infection, Malignancy and Autoimmunity (NIIMA), Universal Scientific Education and Research Network (USERN), Tehran, Iran

**Keywords:** Anthracyclines, Cardiotoxicity, Pediatric cancer, ACE inhibitors, Beta-blockers

## Abstract

**Background:**

Anthracyclines are routinely used in pediatric oncology but cause dose-dependent cardiotoxicity that compromises long-term survival. Neurohormonal agents such as ACE inhibitors (ACEi), angiotensin receptor blockers (ARBs), and beta-blockers have been suggested as preventive or therapeutic agents, yet evidence in children remains limited. Present review aimed to synthesize available data on their efficacy and safety among this population.

**Method:**

A systematic search of PubMed, Scopus, and Web of Science was conducted from inception to August 2025. Eligible studies included pediatric cancer survivors exposed to anthracyclines who received ACEi, ARBs, or beta-blockers with reported cardiac outcomes. Data extraction and risk-of-bias assessments were performed independently by two reviewers. Findings were synthesized narratively owing to study heterogeneity.

**Findings:**

Sixteen studies met inclusion criteria, comprising nine RCTs, three observational studies, two case series, and two case reports. ACEi, mainly enalapril and captopril, were associated with attenuation of left ventricular ejection fraction (LVEF) decline and reduced biomarker elevations, although long-term benefits were inconsistent and side effects such as hypotension were reported. Beta-blockers, particularly carvedilol, improved ventricular function, strain indices, and clinical symptoms in several studies, though the largest trial (PREVENT-HF) did not show significant benefit on primary remodeling outcomes, except in high-risk subgroups. Overall, the evidence suggests that while preventive use of ACEi/ARBs and beta-blockers shows more consistent benefits, findings regarding their role in reversing established cardiotoxicity remain variable and should be interpreted cautiously.

**Conclusion:**

ACEi/ARBs and beta-blockers showed promise in preventing or mitigating anthracycline-induced cardiotoxicity among children, with consistent benefits on surrogate outcomes but uncertain durability and survival impact. Early initiation and targeted use in high-risk patients appear most advantageous, underscoring the need for large biomarker-guided trials to refine prevention strategies.

**Supplementary Information:**

The online version contains supplementary material available at 10.1186/s40959-026-00447-5.

## Introduction

Anthracyclines are a cornerstone of chemotherapy in pediatric oncology, frequently incorporated into treatment regimens for a wide variety of childhood cancers, including leukemia, lymphomas, and solid tumors. These agents, such as doxorubicin and daunorubicin, are known to increase the chances of survival in children with cancer significantly. With every medication, there is a clinical imperative to address its long-term health consequences. Among these, cardiotoxicity is a challenge that can compromise both immediate treatment success and late-life health outcomes in cancer survivors who are treated by anthracyclines [1–3].

Cardiotoxicity, in the context of anthracycline use, refers to damage to cardiac muscle cells leading to impaired myocardial function. The incidence and prevalence of anthracycline-induced cardiotoxicity among pediatric cancer survivors vary across studies. For example, a multiethnic Asian cohort identified about 7% of pediatric patients developing cardiotoxicity, with a subset manifesting clinical heart failure [4]. The variability across tumor types is also notable, with hematologic malignancies such as acute myeloid leukemia (AML) often showing higher cardiotoxicity rates, possibly due to treatment intensiveness and supportive care variances [5, 6].

Clinically, it can manifest as subclinical declines in left ventricular (LV) function, symptomatic heart failure, arrhythmias, or even sudden cardiac death. Cardiac dysfunction, whether acute or chronic, is associated with lower event-free survival (EFS) and overall survival (OS) in pediatric patients, especially in high-risk groups such as children with AML [5]. Beyond early treatment phases, long-term cardiac mortality remains a concern, with survivors exhibiting progressively increased risks of heart failure and cardiac-related death over decades following cancer therapy [3, 7]. Studies reported that cardiotoxicity is dose-dependent, meaning the cumulative dose of anthracyclines that can be safely administered is constrained by the risk of inducing cardiac damage. Thus, balancing the therapeutic benefits of anthracyclines against their potential for life-threatening cardiotoxicity presents a critical clinical dilemma in pediatric oncology [3, 8, 9].

The prevention and treatment of anthracycline cardiotoxicity parallels general heart failure management, including beta-blockers, angiotensin-converting enzyme (ACE) inhibitors, and angiotensin receptor blockers (ARBs). These medications aim to improve cardiac output, reduce remodeling, and mitigate disease progression [10, 11]. However, pediatric-specific data supporting the efficacy and safety of these agents remain limited; thus, here we aim to systematically review the role of ACEi/ARBs and beta-blockers in children’s malignancies treated with anthracyclines.

## Methods

### Eligibility criteria

We included studies evaluating the role of ACE inhibitors, ARBs, or beta-blockers in preventing or treating anthracycline-induced cardiotoxicity among pediatric patients with malignant diseases. Studies were required to report at least one cardiac outcome, including echocardiographic measures, clinical endpoints, or biomarker outcomes. Only English language papers were selected. Besides, no limitation was placed for the study design. We excluded reviews, editorials, animal studies, and studies with mixed adult and pediatric populations unless pediatric data were reported separately.

### Outcome measures

In this review, the term cardioprotection is used to describe pharmacologic interventions, including ACE inhibitors, ARBs, and beta-blockers, that are intended to prevent or mitigate anthracycline-induced cardiac injury and remodeling. This encompasses preservation of LV function, attenuation of biomarker elevations (e.g., troponins, natriuretic peptides), improvement in strain indices, and reduction in the risk of progression to symptomatic heart failure. By defining cardioprotection in this way, we highlight its role in maintaining both subclinical and clinical cardiac health in pediatric cancer survivors exposed to anthracyclines. Further we divded the cardioprotection to primary or secondary levels, in order to indicate the preventive or therapeutic effect of the interventions, respectively. In other words, primary cardioprotection refers to the use of pharmacologic strategies initiated before detectable cardiac dysfunction, with the intent of preventing anthracycline-induced myocardial injury. Secondary cardioprotection refers to the initiation of these same agents after evidence of cardiac dysfunction has emerged, with the goal of halting progression and facilitating recovery.

### Primary cardioprotectionInformation sources

A comprehensive literature search was performed in PubMed, Scopus, and Web of Science from database inception to 16 August 2025. Additional sources, including Google scholar search engine, conference abstracts, and forward and backward citation screening were searched manually, with no restrictions on publication year.

### Search strategy

Database-specific search strategies combined controlled vocabulary and free-text terms related to ACE inhibitors, ARBs, and beta-blockers, pediatric populations, and cancer. The full search strings for each database are provided in **Table ****S1**.

### Study selection

**Fig. 1:**
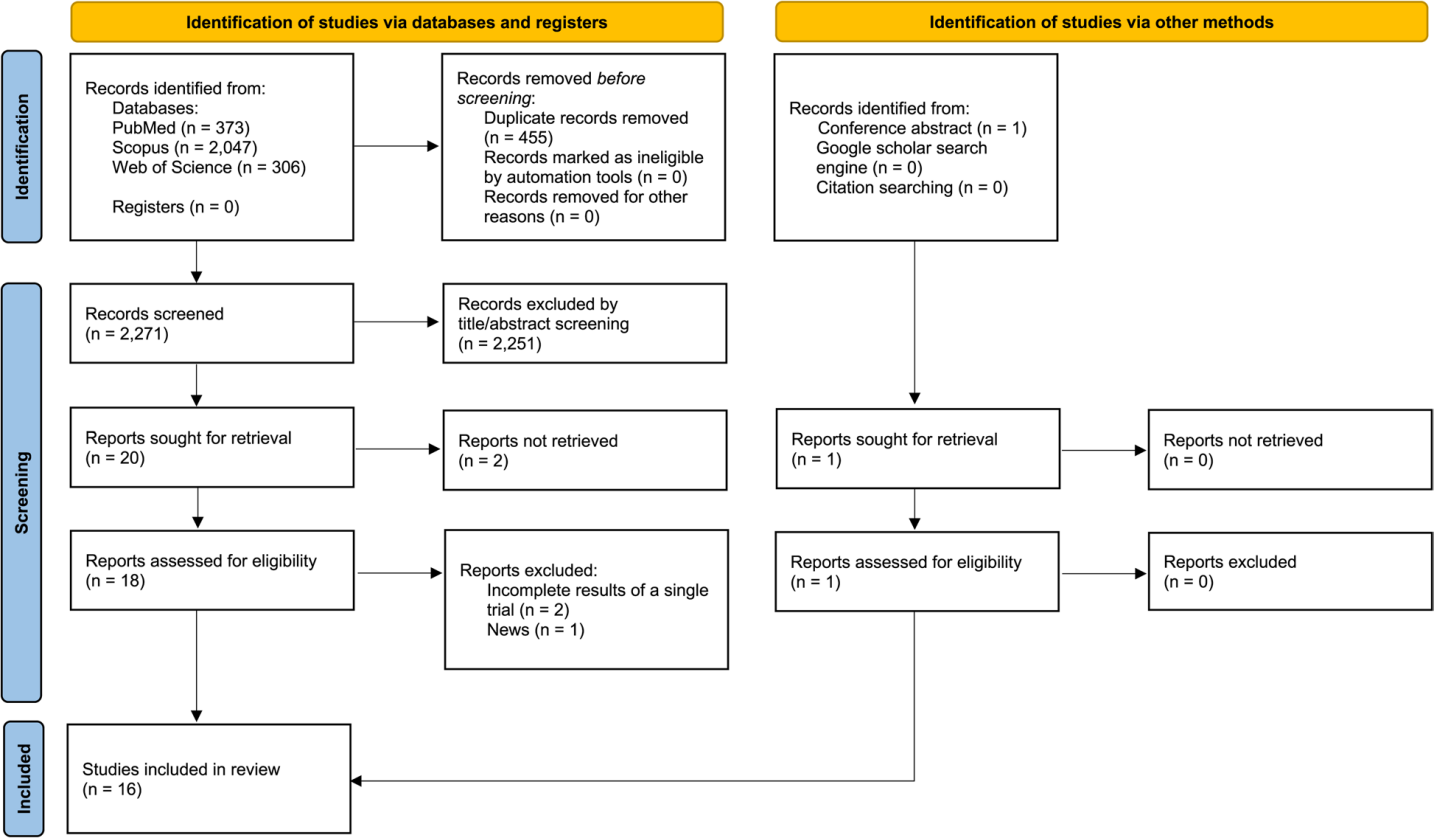
PRISMA flow chart of included studies

All retrieved records were imported into EndNote software, version 20, and duplicates were removed. Two reviewers independently screened titles and abstracts for relevance, followed by full-text review of potentially eligible articles. Disagreements were resolved by consensus. The selection process followed preferred reporting items for systematic reviews and meta-analyses (PRISMA) 2020 guidelines, and the flow of studies is summarized in Fig. 1 [12].

### Data extraction

A standardized data extraction form was developed to collect information on study design, location, recruitment period, eligibility criteria, population characteristics, time of cardiac testing, intervention details (e.g., medication, dose, and duration), comparators, cardiac outcomes, and follow-up duration. Data extraction was performed independently by two reviewers, with discrepancies resolved by discussion.

### Quality assessment methodology

The methodological quality of included studies was evaluated using appropriate critical appraisal tools tailored to each study design. Randomized trials were assessed using the revised Cochrane Risk-of-Bias tool for randomized trials (RoB 2), which evaluates five key domains: randomization process, deviations from intended interventions, missing outcome data, measurement of the outcome, and selection of the reported result. Cohort studies were appraised using the Newcastle-Ottawa Scale (NOS), a validated tool that assigns up to 9 stars across three domains: selection of study groups (0–4 stars), comparability of groups (0–2 stars), and assessment of outcomes (0–3 stars). Case series were evaluated using the Joanna Briggs Institute (JBI) critical appraisal checklist for case series, which comprises 10 items assessing inclusion criteria, measurement standardization, identification methods, participant inclusion completeness, demographic and clinical reporting, outcome documentation, site characteristics, and statistical analysis appropriateness. Case reports were assessed using the JBI critical appraisal checklist for case reports, consisting of eight items evaluating patient demographic description, history presentation, current clinical condition, diagnostic procedures, intervention description, post-intervention condition, adverse event reporting, and educational value. All quality assessments were conducted independently by two reviewers, with disagreements resolved through discussion when necessary.

### Data synthesis

We performed a qualitative synthesis of included studies, structured by intervention class (ACE inhibitors or ARBs vs. beta-blockers). Due to heterogeneity in study designs, patient populations, interventions, and reported outcomes, a meta-analysis was not feasible. Narrative synthesis focused on effects on echocardiographic parameters, clinical outcomes, and biomarkers.

## Results

Following the search of key databased, 2,726 records were identified (PubMed: 373, Scopus: 2,047, and Web of Science: 306), of which 455 duplicates were removed. After screening of 2,271 records through title/abstract, 20 records were candidate for full-text screening. Of these, the full-text of two records was not found. Of the remaining 18 papers, two were the incomplete reports of one of the included studies [13, 14] and one was a news paper [15]. On the other side, searching the conference abstracts resulted in identification of one eligible study [16]. Eventually, 16 papers were included to the current systematic review [16–31] **(**Fig. 1**)**.

### ACE inhibitors and ARBs

Table 1 summarize the baseline characteristics of studies that used ACE inhibitors and/or ARBs for managing cardiotoxicity following anthracycline therapy among childhood cancer survivors.

**Table 1:** The baseline characteristics of included studies examining the role of ACE inhibitors or ARBs in children treated with anthracyclines

First author (Year)	Type of paper	Location	Recruitment period	Design	Eligibility criteria		Cardiac testing time	Medications		No of patients			Age at diagnosis		Age at baseline			Sex			Follow-up period	Outcomes
					Inclusion criteria	Exclusion criteria		Int	Ctrl	Int	Ctrl	Int		Ctrl		Int	Ctrl		Int	Ctrl		
Primary cardioprotection																						
Selim et al. (2025)[16]	Conference abstract	Egypt	-	Randomized clinical trial	Newly diagnosed malignant bone tumor pediatric patients	-	-	Prophylactic ACE inhibitors from day one of chemotherapy	Placebo	50	61	Median: 13.2 years		Median: 10.6 years		-	-		-	-	12 months	Primary outcomes: 1) ≥ 10% reduction in LVEF 2) ≥ 15% decrease in GLS within 12 months’ post-treatment Secondary outcomes: Death or hospitalization due to cardiac events
Gupta et al. [21]	Full-paper	India	August 1, 2014, to September 30, 2016,	Randomized, double-blind, placebo-controlled trial	1) Age 2–16 years at the time of diagnosis 2) Confirmed diagnosis of ALL/lymphoma (Hodgkin and non-Hodgkin lymphoma) 3) Estimated cumulative anthracycline dose ≥ 200 mg/m^2^	1) Relapsed disease 2) Previously treated (defaulter/irregular treatment) 3) Resting EF < 50% or FS < 25% 4) Preexisting cardiac disease (valvular disease, cardiomyopathy) 5) Significant renal disease (serum Cr > 3 times normal and/or estimated GFR < 30 mL/min/m2) 6) Significant hepatic disease (> 3 times ALT/AST)	At baseline and after 6 months	Enalapril tab in a dosage of 0.1 mg/kg/day once a day from the first day of chemotherapy for 6 months	Placebo	44	40	-		-		Mean (SD): 8.8 (3.1)	Mean (SD): 8.8 (2.9)		M: 31/F: 13	M: 30/F: 10	6 months	Primary Outcome: Incidence of cardiotoxicity as defined by a ≥ 20% decrease in LVEF from baseline to 6 months Secondary Outcomes: 1) Change in LVEF over 6 months 2) Levels of cardiac biomarkers at baseline and after 6 months: cTnI, proBNP, and CK-MB 3) Number of patients with proBNP levels ≥ 100 pg/mL at 6 months 4) Development of heart failure or arrhythmias during the study period
**Secondary cardioprotection**																						
Silber et al. (2004)[30]	Full-paper	USA	October 1994, to March 1999	Randomized, double-blind, controlled clinical trial	1) Patients aged 8 years and older 2) Developed cancer before the age of 20 years 3) Had been treated with anthracyclines 4) Had been at least 4 years from diagnosis and 2 years from completing all cancer treatment 5) One or more of the following measures of decline in cardiac systolic performance: an echocardiographic LVFS of ≤ 29%, or a 10% decrease from an earlier test; a LVEF of ≤ 55%, reduction in EF with exercise, or a 10% decrease in resting EF over time; a MCI of ≤ 7.4 L/min/m2 on cycle ergometry at peak exercise; or an ECG QTc interval of ≥ 440 milliseconds on resting ECG	1) Had been treated with or were currently being treated with an ACE inhibitor 2) Were receiving digoxin or other inotropic agents 3) Had a history of congestive heart failure 4) Had a history of congenital heart disease that study investigators believed could account for the abnormal cardiac findings or that would impair any potential benefit from enalapril 5) Had documented renal artery stenosis 6) Were females planning to become pregnant during the study period	Every 6 months	Enalapril in a dosage of 0.05 mg/kg/day, progressively escalated to 0.10 mg/kg/day, and finally received 0.15 mg/kg/day if no side effects were noted	Placebo	69	66	Median (range): 7.2 years (3-21.8)		Median (range): 8.2 years (0.3.19.3)		Median (range): 17 years (8.3–31.5)	Median (range): 18.9 years (8.1–30.6)		M: 37/F: 32	M: 41/F: 25	Mean (range): 2.889 years (2 weeks-6.1 years)	Primary outcome: MCI Secondary outcomes: 1) LVESWS 2) FS 3) SVI 4) Adverse events
Mandric et al. (2008)[26]	Conference abstract	Romania	-	Controlled clinical trial	1) Survivors of pediatric cancer 2) Treated with certain therapeutical protocols for hematological malignancies, which included anthracyclines- doxorubicin 3) Had at least one cardiac abnormality identified at any time after anthracycline exposure	-	At baseline and at 3, 6, 12, and 16 months after initiation of enalapril/placebo therapy	Enalapril (dose range between 0.2–0.5 mg/kg/day)	Placebo	10	20	Mean: 6 years		-		Range: 6–14 years	-		-	-	16 months	1) LVESD and LVEDD 2) FS 3) LV mass 4) LV posterior wall thickening 5) Interventricular septal thickening 6) Tei index
Harrington et al. (2018)[22]	Full-paper	USA	Between 2006 and 2015	Retrospective study	1) Were exposed to anthracycline-containing chemotherapy at ≤ 21 years of age 2) Had echocardiograms performed before and during treatment with ACEi/ARB that were of adequate quality to perform speckle tracking analysis 3) Started ACEi/ARB at the discretion of the treating physician as therapy for post-chemotherapy cardiac dysfunction (abnormal LV function assessment or LV dilation) and/or hypertension	-	At the initiation of chemotherapy, after chemotherapy, at the initiation of ACEi/ARB, and after initiation of ACEi/ARB	Most patients were treated with lisinopril (*n* = 17), four were treated with enalapril, and one was treated with losartan after intolerance of an ACE inhibitor	-	22	-	Median (range): 11.1 years (1.7–21.5)		-		-	-		M:9 /F: 13	-	Median (range): 599 days (518–1317)	Primary outcomes: 1) GLS 2) GCS Secondary outcome: 1) LSR 2) CSR
Lipshultz et al. (2002)[24]	Full-paper	USA	From 1984 through 1989	Retrospective study	Patients diagnosed with childhood malignancy from 1984 through 1989 and treated with doxorubicin who had completed treatment at least 1 year before the study began and had received enalapril as of 1990	-	At six time points: 1) The earliest echocardiogram performed at least 1 year after doxorubicin therapy was completed 2) Immediately before enalapril was started 3) The first follow-up study on enalapril (mean duration of enalapril therapy, 0.45 year) 4) The latest follow-up study on enalapril before 1992 (mean duration of enalapril therapy, 2.4 years) 5) The latest follow-up study before 1996 (mean duration of enalapril therapy, 6 years); 6) The latest follow-up study before April 2001 (mean duration of enalapril therapy, 10 years).	Enalapril with a mean dose of 18 mg/day (5–40)	-	18	-	Mean (range): 8.0 years (1-18.1)		-		-	-		M: 9/F: 9	-	10 years	Primary Outcomes: 1) FS 2) LV contractility (SVI) 3) LV end-diastolic posterior wall thickness 4) LVESWS (LV afterload) 5) LV mass 6) Blood pressure (systolic and diastolic) 7) LVEDD Secondary Outcomes: 1) Development of congestive heart failure 2) Need for cardiac transplantation 3) Cardiac-related mortality 4) Changes in echocardiographic parameters over time 5) Adverse event
Hauser and Wilson (2000)[23]	Full-paper	UK	-	Case-series	1) Children with leukemia who had received chemotherapy within the last 10 years 2) Had daunorubicin-induced cardiomyopathy	-	At the end of chemotherapy, at initiation of ACE inhibitor therapy, and during ACE inhibitor treatment	Captopril with a dose of 2–5 mg/kg/day	-	7	-	Median (range): 5.6 years (3.8–7.5)				-			-	-	1 year	FS and EF as indicators of LV function
Tony et al. (2021)[31]	Full-paper	Oman	-	Case-report	-	-	At baseline, before chemotherapy initiation and during hospitalizations	Captopril and Lisinopril	-	1	-	2.5 years		-		2.5 years	-		M: 1	-	Five years	LVEF and CK-MB
Lo et al. (2021) [25]	Full-paper	Taiwan	-	Case-report	-	-	With every episodes of heart failure	Valsartan/sacubitril with the dosage of 0.8 mg/kg/dose twice daily	-	1	-	1 year				7 years			F: 1	-	Two years	LVEF, serum BNP, and clinical manifestations

*ACEi/ARB* Angiotensin-converting enzyme inhibitors/angiotensin receptor blockers, *ECG* Electrocardiogram, *LV* Left ventricle, *LVEF* Left ventricular ejection fraction, *EF* Ejection fraction, *LVFS* Left ventricular fractional shortening, *FS* Fractional shortening, *SVI* Stress-velocity index, *LVESWS,* Left ventricular end-systolic wall stress, *LVESD* Left ventricular end-systolic dimension, *LVEDD* Left ventricular end-diastolic dimension, *GLS* Global peak longitudinal strain, *GCS* Global peak circumferential strain, *LSR* Longitudinal strain rate, *CSR* Circumferential strain rate, *cTnI* Cardiac troponin I, *proBNP* Pro-B-type natriuretic peptide, *CK-MB* Creatine kinase MB, *MCI* Maximal cardiac index, *GFR* Glomerular filtration rate, *Cr* Creatinine, *ALT* Alanine aminotransferase, *AST* Aspartate aminotransferase, *ALL* Acute lymphoblastic leukemia, *SD* Standard deviation, *M* Male, *F* Female

#### Primary cardioprotection

Selim and colleagues [16] evaluated the cardioprotective efficacy of ACE inhibitors in pediatric patients with malignant bone tumors, including osteosarcoma and Ewing sarcoma, who were treated with anthracycline-based chemotherapy. A total of 111 newly diagnosed patients were randomized to either receive prophylactic ACE inhibitors from the initiation of chemotherapy (n = 50) or to a control group without ACE inhibitor therapy (n = 61). The follow-up time was 12 months. The intervention group demonstrated a lower incidence of ≥ 10% left ventricular ejection fraction (LVEF) reduction (50% vs. 67% in controls) and superior EFS based on LVEF, with a longer median survival duration (13.80 vs. 7.82 months) and a higher 12-month survival probability (54% vs. 34%, P = 0.03). Cumulative incidence analysis, demonstrated consistent findings with the survival analysis, yielding a comparable p-value (P = 0.03). However, when age and body surface area were included as covariates in a multivariable Cox regression model, the protective effect was attenuated and no longer statistically significant (Hazard ratio = 0.60, 95% CI: 0.36 to 1.00, P = 0.051), suggesting that part of the observed benefit may be explained by baseline differences between groups. Global peak longitudinal strain (GLS)-based analyses did not reveal significant differences between groups, with 12-month EFS of 54% vs. 47% and cumulative incidence of 45.5% vs. 52.9% in the intervention and control groups, respectively. Importantly, no life-threatening cardiac events occurred in either arm, aside from one case of cardiogenic shock in the intervention cohort. In conclusion, prophylactic administration of ACE inhibitors demonstrated a potential cardioprotective signal with improvements in LVEF-related outcomes, though statistical significance was not consistently achieved [16].

Besides, a randomized, double-blind, placebo-controlled trial was conducted by Gupta et al. [21] to evaluate the role of enalapril in the incidence of anthracycline-induced cardiotoxicity in children with leukemia and lymphoma. Eighty-four patients with no history of cardiac diseases, receiving anthracyclines were randomized into two groups: enalapril (n = 44) and placebo (n = 40). The study interventions were started at the same time with starting chemotherapy and continued for 6 months. A ≥20% decrease in LVEF was observed in three patients in the placebo group but none in the enalapril group (P = 0.21). LVEF decreased significantly in both groups, but more in the placebo group (LVEF at 6 months: 62.25 ± 5.49 vs. 56.15 ± 4.79, P < 0.001). Cardiac biomarkers increased more in the placebo group, with a significant rise in pro B-type natriuretic peptide (BNP) (49.60 ± 35.97 vs. 98.60 ± 54.24, P < 0.001) and cardiac troponin I (cTnI) (0.01 ± 0.00 vs. 0.011 ± 0.003, P = 0.035), but no significant difference in creatine kinase MB (CK-MB) (1.08 ± 0.18 vs. 1.21 ± 0.44, P = 0.079). Among enalapril patients, 9.1% presented with proBNP levels ≥100 pg/mL, compared to 37.5% in the placebo group (P < 0.001). No cases of heart failure or arrhythmias were reported. The findings suggest that enalapril may have a protective effect against early anthracycline-induced cardiotoxicity [21].

#### Secondary cardioprotection

Silber and colleagues [30] conducted a randomized, double-blind, placebo-controlled clinical trial investigating whether the ACE inhibitor, enalapril, could prevent cardiac function deterioration in long-term pediatric cancer survivors exposed to anthracycline. The included patients were at least 8 years of age or older who developed cancer before the age of 20, had been treated with anthracyclines, and had been at least 2 years from completing all cancer treatment. Survivors of pediatric cancer must have had at least one cardiac abnormality identified at any time after anthracycline exposure. The study included 135 patients, randomly assigned to receive either enalapril or a placebo. The study found no significant difference in the rate of change in maximal cardiac index (MCI) per year between the enalapril and placebo groups (*P* = 0.36), indicating that enalapril did not improve exercise capacity. However, enalapril significantly reduced left ventricular end-systolic wall stress (LVESWS) during the first year of treatment (*P* = 0.036). This reduction was maintained over the study period, however no significant change was noted during second year and later (*P* = 0.56). Overall, a 9% decrease in LVESWS by year five was recorded in the enalapril group compared to a 2% increase in the placebo group. The study found no significant difference in fractional shortening (FS) or contractile state, as measured by the stress-velocity index (SVI), between the enalapril and placebo groups over time. Six out of seven patients who experienced clinically significant cardiac deterioration were in the placebo group (*P* = 0.059), suggesting a possible protective effect. Side effects were more common in the enalapril group, with 22% experiencing dizziness/hypotension (vs. 3% placebo, *P* < 0.001) and 10.1% reporting fatigue (vs. 0%, *P* = 0.013). As a result, any theoretical benefits of LVESWS reduction in anthracycline-exposed populations must be weighed against potential side effects from ACE inhibitors when making treatment decisions [30].

The abstract by Mandric and colleagues [26] reported a preliminary prospective clinical study evaluating the effect of enalapril on anthracycline-induced cardiotoxicity in pediatric cancer survivors. Thirty children aged 6 to 14 years, all of whom had received anthracyclines as part of their treatment for hematologic malignancies and subsequently developed at least one cardiac abnormality, were enrolled. The cohort was divided into two groups: ten children received enalapril at a dose of 0.2–0.5 mg/kg/day, while twenty served as controls without enalapril treatment. Echocardiographic assessments were performed at baseline and during follow-up at 3, 6, 12, and 16 months. Over the course of 16 months, the enalapril group demonstrated progressive improvements in several cardiac parameters, including LV dimensions, FS, LV mass, posterior wall thickening, septal thickening, and Tei index, whereas the control group either remained stable or worsened. Therefore, enalapril improved echocardiographic changes associated with anthracycline-induced cardiotoxicity and suggested its early introduction in affected children [26].

Harrington and colleagues [22] designed a retrospective cohort study evaluating the effects of ACE inhibitors and ARBs on cardiac function in childhood cancer survivors using two-dimensional speckle tracking echocardiography (STE). Twenty-two patients were included. The median time from completion of chemotherapy to initiation of ACEi/ARB was 1.9 years and seven patients started ACEi/ARB while they were still receiving chemotherapy. The majority of patients were treated with lisinopril (*n* = 17), four with enalapril, and one with losartan after intolerance of an ACE inhibitor. The mean GLS (*P* < 0.001), global peak circumferential strain (GCS) (*P* < 0.001), and longitudinal strain rate (LSR) (*P* = 0.02) all significantly worsened after chemotherapy. After the median time of 41 days on ACEi/ARB, the mean GLS (*P* = 0.002), GCS (*P* = 0.027), LSR (*P* = 0.022), and circumferential strain rate (CSR) (*P* = 0.027) all significantly improved. Furthermore, there was significant improvement in GLS on ACEi/ARB seen early in therapy (< 1 year on ACEi/ARB, *P* = 0.027) and no additional improvement in GLS after one year of therapy was noted, while the initial improvement was maintained (*P* = 0.023). Besides, the ejection fraction (EF) (*P* = 0.034) and FS (*P* = 0.001) both improved on treatment with ACEi/ARB. The EF before initiating ACEi/ARB was correlated with the change in GLS on therapy (*P* = 0.006), with a worse EF before ACEi/ARB was associated with a greater improvement in GLS on treatment. These findings support the use of ACEi/ARB to treat post-chemotherapy-related cardiovascular changes in childhood cancer survivors [22].

Lipshultz and colleagues [24] conducted a retrospective study on 18 childhood cancer survivors treated with doxorubicin who later received enalapril for LV dysfunction at least one year after chemotherapy was completed. The mean time between the completion of doxorubicin therapy and the start of enalapril was 7 years, with a median follow-up duration of 10 years. Over the first 6 years of enalapril therapy, there was progressive improvement in LV dimension, afterload, FS, and LV mass. However, these parameters deteriorated toward baseline values between 6 and 10 years. Systolic blood pressure decreased significantly during enalapril therapy (*P* < 0.001), while diastolic blood pressure showed a slight downward trend (*P* = 0.07). By 6 years of enalapril therapy, all six patients who were symptomatic at baseline had either died or undergone cardiac transplantation, compared with three of the 12 asymptomatic patients. At 10 years, four of the remaining nine patients developed heart failure. Over the first 2.4 years of enalapril treatment, symptomatic patients showed significantly greater LV dilation (*P* = 0.02) but greater improvement in contractility (*P* = 0.04) than asymptomatic patients. Survival analyses showed that time to dropout (death or transplantation) was significantly shorter in patients who were older at enalapril initiation (*P* = 0.01), older at cancer diagnosis (*P* = 0.006), had a shorter time interval from doxorubicin to enalapril (*P* = 0.003), or had more abnormal LV dimensions at baseline (*P* = 0.009). Finally, Higher baseline systolic blood pressure was significantly correlated with lower LV afterload at follow-up (*P* = 0.04), while higher baseline diastolic blood pressure predicted multiple favorable outcomes, including reduced LV dimension (*P* = 0.02), reduced LV afterload (*P* = 0.02), lower LV mass (*P* = 0.03), and improved LV contractility (*P* = 0.04) and FS (*P* < 0.001). No adverse enalapril reactions were noted. Thus, among doxorubicin-exposed childhood cancer survivors, baseline diastolic blood pressure was the most powerful predictor of response to enalapril. However, despite these transient benefits, progressive LV wall thinning and eventual clinical deterioration persisted, underscoring the limited long-term efficacy of enalapril in preventing adverse cardiac outcomes in this population [24].

The article by Hauser and Wilson [23] reported the use of ACE inhibitors in children who developed anthracycline-induced cardiomyopathy. The study describes seven pediatric patients (five with AML and two with acute lymphoblastic leukemia [ALL]) who had received daunorubicin as part of their chemotherapy and subsequently developed congestive heart failure within a median of 3.8 years after completing treatment. All children showed markedly impaired cardiac function, with low FS and EF, enlarged ventricular dimensions, and significant mitral regurgitation. All patients were treated with captopril at 2–5 mg/kg/day in addition to conventional therapy with digoxin and diuretics. In six of the seven children, FS and EF normalized within a median of 7.9 months. In the remaining patient, function improved but did not return fully to normal by the time of reporting. In four children, ACE inhibitor therapy was later discontinued after approximately 10.4 months of captopril administration, and normal cardiac function persisted at one-year follow-up. Therefore, in this study ACE inhibitor successfully reverse anthracycline-induced cardiomyopathy in children and suggest that early intervention, even in asymptomatic patients with evidence of myocardial damage, might help preserve cardiac function [23].

The article by Tony et al. [31] presents the case of a 2.5-year-old Omani boy with AML who developed acute anthracycline-related cardiotoxicity after treatment with daunorubicin. At the start of chemotherapy, echocardiography showed a structurally normal heart with preserved systolic function and a LVEF of 55–60%. Shortly after the second dose of daunorubicin, the patient experienced acute cardiac decompensation characterized by tachycardia, respiratory distress, seizure, bradycardia, and hypotension. Cardiac investigations revealed a fall in LVEF to 35% and elevated cardiac biomarkers, including CK-MB and cTnI. The patient was started on diuretics, captopril as an ACE inhibitor, spironolactone, and later digoxin. During follow-up, captopril was replaced with lisinopril. With this regimen, the patient gradually recovered, and cardiac function improved and stabilized. Over the course of five years of follow-up, his LVEF returned to around 55% and remained stable. He remained in remission from AML and continued on lisinopril for long-term cardiac support. In conclusion, the use of ACE inhibitors was considered crucial in the patient’s recovery and long-term cardiac stability [31].

The article by Lo et al. [25] was a case-report describing the use of low-dose valsartan/sacubitril (an angiotensin receptor-neprilysin inhibitor, ARNI) in a pediatric patient with chemotherapy-induced cardiomyopathy and acute decompensated heart failure. The patient was a 7-year-old girl with a history of AML treated with intensive chemotherapy, including anthracyclines, followed by stem cell transplantation. Although her cardiac function was initially preserved, she later developed dilated cardiomyopathy and progressive heart failure. The dilated cardiomyopathy was well controlled by captopril (1 mg/kg/day) with stable LVEF of 50% for about 2 years. However, after that, acute heart failure developed which primarily improved with multiple anti-congestive medications. She experienced repeated episodes of acute decompensated heart failure with markedly reduced LVEF (~ 19–22%). During her second episode of decompensation, conventional management including diuretics, beta-blocker, spironolactone, ramipril, and inotropes failed to stabilize her condition. At this stage, ramipril was replaced with valsartan/sacubitril at a starting dose of 0.8 mg/kg twice daily, which was lower than guideline-recommended pediatric doses to avoid hypotension. Within days of ARNI initiation, urine output increased, BNP levels decreased, pleural effusion resolved, and her hemodynamic status improved. Over the following weeks, her chest X-ray showed reduced cardiomegaly, and her LVEF improved to 35.4%. At one-year follow-up, her LVEF had normalized to 56.5%, and she was asymptomatic (i.e., New York Heart Association [NYHA] class I) without significant adverse effects. As a result, valsartan/sacubitril appeared effective at a dose lower than the recommended pediatric starting dose, suggesting potential for efficacy with reduced risk of hypotension. Although adult guidelines recommend ARNI use primarily in stable chronic heart failure, in this case the drug was effective in acute decompensated pediatric heart failure refractory to standard therapy [25].

### Beta-blockers

Table 2 summarize the baseline characteristics of studies that used beta-blockers for managing cardiotoxicity following anthracycline therapy among childhood cancer survivors.

**Table 2:** The baseline characteristics of included studies examining the role of beta-blockers in children with cancer treated with anthracyclines

First author	Article type	Location	Recruitment period	Design	Eligibility criteria		Cardiac testing time	Medications			No of patients			Age in diagnosis			Age at baseline		Sex		Follow-up period	Outcomes
					Inclusion criteria	Exclusion criteria		Int	Ctrl	Int		Ctrl	Int		Ctrl	Int		Ctrl	Int	Ctrl		
Primary cardioprotection																						
El-Shitany et al. (2012)[20]	Full-paper	Egypt	March 2008 to March 2010	Parallel-assignment, randomized, clinical trial	1) Children 6–12 years old 2) Newly diagnosed with ALL confirmed by complete blood picture, bone marrow examination, immunophenotyping assessed by flow cytometry, and FISH technique	1) Previous chemotherapy or radiotherapy 2) Presence of any cardiac disease, either congenital or acquired 3) Any cardiac lesion detected in baseline echocardiography 4) Any associated systemic disease, such as renal dysfunctions and hepatocellular insufficiency 5) Medications affecting cardiac function, including ACEi/ARB, diuretics, and other beta-blockers	Conventional 2D, PTD, and 2DS echocardiography was done before and after carvedilol therapy	Carvedilol was administered for 5 days before every dose of doxorubicin. The starting carvedilol dose was 0.1 mg/kg/day in 2 divided doses, increased at weekly intervals until reaching a dose of 1 mg/kg before the last dose of doxorubicin	Placebo	25		25	Mean (SD): 8.5 years (2.9)		Mean (SD): 9.5 years (2.6)	Mean (SD): 8.5 years (2.9)		Mean (SD): 9.5 years (2.6)	M: 11/F: 14	M: 12/F: 13	One week after the last doxorubicin dose	Primary outcomes: 1) FS 2) GPSS 3) LV Diastolic Function: E/A ratio Secondary outcomes: 1) PTD Parameters: s, e/a ratio 2) Biomarkers of Cardiac Injury: Plasma cTnI levels, Plasma LDH levels, Plasma CPK levels
**Primary and secondary cardioprotection**																						
Seth et al. (2023)[28]	Conference abstract	India	-	Clinical trial	Pediatric patients of acute leukemia suffering from chemotherapy induced cardiotoxicity Preventive arm: Cardiac dysfunction as a fall in EF by 10% Therapeutic arm: Cardiac dysfunction as a fall in EF below 50%	-	Before each chemotherapy cycle	ACE inhibitors and Beta blockers in both groups	-	Preventive arm: 12 Therapeutic arm: 26		-	-		-	-		-	-	-	-	LVEF and NT-proBNP
**Secondary cardioprotection**																						
Armenian et al. (2024)[18]	Full-paper	USA and Canada	July 3, 2012, to June 22, 2020	Multicenter, randomized phase IIb, double-blinded, placebo-controlled trial	1) Diagnosis of cancer at age ≤ 21 years, regardless of age at enrollment 2) Lifetime anthracycline dose ≥ 250 mg/m² without the use of dexrazoxane cardioprotection 3) ≥ 2 years from completion of cancer treatment 4) Weight ≥ 40 kg at study enrollment	1) Patients receiving treatment for cardiomyopathy or heart failure 2) Had an EF < 50% by radionuclide angiogram or echocardiogram, or FS < 25% by echocardiogram 3) Patients with bilirubin, or AST, or ALT ≥3x the upper limit of the institutional norm	At baseline (t0), 6 months (t1), 1 year (t2), 18 months (t3), and 2 years (t4)	Oral tablets 6.25 mg/twice daily for two years	Placebo	89		93	Median (IQR): 11.0 years (6.5–15.2)		Median (IQR): 11.7 years (5.8–15.6)	Median (IQR): 23·2 years (19.6–34.0)		Median (IQR): 25.2 years (20.2–36.6)	M: 46/F: 43	M: 45/F: 48	Median (IQR): 725 days (378–730)	Primary outcome: Echocardiogram-derived LVWT/D z-score (LVWT/Dz) Secondary outcomes: 1) LVESD, LVEDD, LVESV, and LVEDV 2) LVESWS 3) LV mass 4) EF 5) FS 6) Diastolic function (E/A wave ratio) 7) Cardiac blood biomarkers (BNP, NT-proBNP, Troponin-I, and Galectin-3) 8) Adverse events
Armenian et al. (2024)[19]	Full-paper	USA and Canada	July 3, 2012, to June 22, 2020	Multicenter, randomized phase IIb, double-blinded, placebo-controlled trial	1) Elevated baseline NT-proBNP (i.e., age and sex-specific > 97.5th percentile) 2) Diagnosis of cancer at age ≤ 21 years, regardless of age at enrollment 3) Lifetime anthracycline dose ≥ 250 mg/m² without the use of dexrazoxane cardioprotection 4) ≥ 2 years from completion of cancer treatment 5) Weight ≥ 40 kg at study enrollment	1) Patients receiving treatment for cardiomyopathy or heart failure 2) Had an EF < 50% by radionuclide angiogram or echocardiogram, or FS < 25% by echocardiogram 3) Patients with bilirubin, or AST, or ALT ≥3x the upper limit of the institutional norm	At baseline (t0), 6 months (t1), 1 year (t2), 18 months (t3), and 2 years (t4)	Oral tablets 6.25 mg/twice daily for two years	Placebo	16		16	-		-	Mean ± SD: 34.0 years ± 9.8			M: 22/F: 10		Median (IQR): 725 days (378–730)	Primary outcome: Echocardiogram-derived LVWT/D z-score (LVWT/Dz) Secondary outcomes: 1) LVESD, LVEDD, LVESV, and LVEDV 2) LVESWS 3) LV mass 4) EF 5) FS 6) Diastolic function (E/A wave ratio) 7) Cardiac blood biomarkers (BNP, NT-proBNP, Troponin-I, and Galectin-3) 8) Adverse events
Agarwal et al. (2005)[17]	Conference abstract	USA	July 2000 to January 2005	Controlled clinical trial	Pediatric oncology patients suffering from early or late onset cardiomyopathy following anthracycline therapy	-	At cardiomyopathy diagnosis and during follow-up	Carvedilol initiation dose of 3.25 mg twice daily or 0.025 mg/Kg per dose twice daily and uptitrated to maximum tolerated doses	No treatment	21		13	Mean (range): 11.8 years (6 months-24 years)		-	-		-	-	-	-	LVEF, LVFS, and NYHA classification
Pozza et al. (2025)[27]	Conference abstract	Italy	-	Retrospective study	1) Pediatric patients affected with ALL 2) Were treated according to AIEOP-BFM ALL 2009 or AIEOP-BFM ALL 2017 3) Disease onset 2018–2021	-	At baseline (T0), before each anthracycline cycle and at stop therapy	Carvedilol	No treatment	24		12	-		-	Mean ± SD: 9.4 years ± 5.2			M: 42/F: 24		-	GLS
Shaddy et al. (1995)[29]	Full-paper	USA	-	Case-series	Pediatric patients who had symptoms of severe congestive heart failure after receiving doxorubicin as cancer chemotherapy	-	Before metoprolol initiation and after two months	Metoprolol 6.25 mg twice daily and up-titrated to individualized maximum tolerated doses (75–125 mg/day)	-	3		-	2, 3.5, 7.5 years		-	15, 16, 12 years		-	-	-	2 months	LVEF, LVFS, and NYHA classification

ACEi/ARB Angiotensin-converting enzyme inhibitors/angiotensin receptor blockers, *LV* Left ventricle, *LVEF* Left ventricular ejection fraction, *EF* Ejection fraction, *LVFS* Left ventricular fractional shortening, *FS* Fractional shortening, *LVESWS* Left ventricular end-systolic wall stress, *LVESD* Left ventricular end-systolic dimension, *LVEDD* Left ventricular end-diastolic dimension, *LVESV* Left ventricular end-systolic volume, *LVEDV* Left ventricular end-diastolic volume, *GLS* Global peak longitudinal strain, *GPSS* Global peak-systolic strain, *E* Mitral flow early-phase filling velocity, *A* Peak atrial-phase filling velocity, *s* Tissue doppler peak mitral annulus systolic velocity, *e* Tissue doppler mitral flow early-phase filling velocity, *a* Tissue doppler peak atrial-phase filling velocity, *cTnI* Cardiac troponin I, *BNP* B-type natriuretic peptide, *NT-proBNP* N-terminal pro-BNP, *ALT* Alanine aminotransferase, *AST* Aspartate aminotransferase, *LDH* Lactic dehydrogenase, *CPK* Creatine phosphokinase, *ALL* Acute lymphoblastic leukemia, *FISH* Fluorescence in situ hybridization, *PTD* Pulsed tissue doppler, *2D* Conventional 2-dimensional echocardiography, *2DS* 2-dimensional longitudinal strain echocardiography, *NYHA* New York heart association, *IQR* Interquartile range, *SD* Standard deviation, *M* Male, *F* Female

#### Primary cardioprotection

The study by El-Shitany et al. [20] was a clinical trial investigating the protective effects of carvedilol on doxorubicin-induced cardiotoxicity in children with ALL. Fifty children, aged 6–12, who were newly diagnosed with ALL were randomly assigned to two equal groups: one receiving doxorubicin alone and the other receiving carvedilol five days before doxorubicin. One week after the last doxorubicin dose, doxorubicin alone caused a 16.25% decrease in FS (*P* = 0.01), a 37.15% decrease in apical long axis (ALX) (*P* < 0.001), and 19% decrease in global peak-systolic strain (GPSS) (*P* < 0.001) compared with the values of before doxorubicin treatment, indicating LV systolic dysfunction. However, pretreatment with carvedilol 14.9% improved FS (*P* = 0.0015), 56.6% improved ALX (*P* < 0.001), and 34.7% improved GPSS (*P* < 0.001) compared to the doxorubicin-only group. Additionally, doxorubicin 100% increased plasma cTnI levels (*P* = 0.005) and 156% increased lactic dehydrogenase (LDH) (*P* = 0.006), markers of cardiac injury. Carvedilol pretreatment inhibited this increase, leading to significantly lower levels of cTnI (62%, *P* = 0.0008) and LDH (57%, *P* = 0.0001) compared to the doxorubicin-only group. Diastolic function parameters (E, A, and E/A) showed no significant changes in either group. Similarly, pulsed tissue doppler (PTD)-derived parameters (s, e, and a velocities) were not significantly different post-treatment. These results supports the protective role of carvedilol against doxorubicin-induced cardiotoxicity [20].

#### Primary and secondary cardioprotection

The prospective study by Seth et al. [28] investigated the role of combined ACE inhibitor and beta-adrenergic blockade in mitigating early chemotherapy-induced cardiotoxicity among pediatric patients with acute leukemia. A total of 186 children underwent serial assessment of LV function using echocardiography and biomarker surveillance prior to each chemotherapy cycle. Patients exhibiting either a ≥ 10% decline in LVEF from baseline (preventive cohort, *n* = 12) or an absolute reduction of LVEF below 50% (therapeutic cohort, *n* = 26) were initiated on ACE inhibitors and beta-blockers. In the preventive cohort, subclinical dysfunction was often heralded by N-terminal pro-BNP (NT-proBNP) elevation, with subsequent modest reductions in LVEF (mean 48.2%). Cardioprotective therapy facilitated full recovery of ventricular function without disruption of chemotherapy in all patients in the protective arm. In the therapeutic cohort, mean LVEF declined to 40.5%, necessitating temporary interruption of chemotherapy and recovery of ventricular function was achieved in all but one individual. Notably, troponin elevations were not observed in either cohort. Hence, the early anthracycline cardiotoxicity in pediatric leukemia is largely reversible when detected through vigilant surveillance and promptly treated with ACE inhibitors and beta-blockades [28].

#### Secondary cardioprotection

Armenian et al. [18] conducted a randomized, double-blind, placebo-controlled phase IIb trial (PREVENT-HF) at 30 hospitals in the United States and Canada to evaluate the effects of low-dose carvedilol on heart failure risk in childhood cancer survivors with high-dose anthracycline exposure. A total of 182 participants enrolled (carvedilol: *n* = 89; placebo: *n* = 93). The median follow-up was 725 days. After two years, no significant difference between the carvedilol and placebo groups was observed in the left ventricular wall thickness-dimension ratio Z score (LVWT/Dz), an established marker of adverse cardiac remodeling in survivors (*P* = 0.14). However, carvedilol significantly reduced LVESWS compared to placebo (-7.73, 95% CI: -14.40 to -1.06, *P* = 0.023). No significant differences were found in EF (*P* = 0.33), FS (*P* = 0.93), or blood biomarkers, including BNP (*P* = 0.18), NT-proBNP (*P* = 0.50), Galectin-3 (*P* = 0.93), and cTnI (*P* = 0.46). Eight participants developed cardiac events during the two years, with six occurring in the placebo group. A post-hoc analysis in highly treatment-adherent participants (≥ 92% adherence) indicated a lower cardiac event rate in the carvedilol group (*P* = 0.052). Two grades 2 adverse events (shortness of breath and arthralgia) were reported in the carvedilol group, with no grades ≥ 3 adverse events or deaths. The study concluded that while carvedilol was safe, it did not significantly improve LVWT/Dz, and further research is needed to assess its potential benefits in high-risk childhood cancer survivors [18].

A post hoc analysis of PREVENT-HF trial was conducted to examine the efficacy of carvedilol on cardiac remodeling in childhood cancer survivors exposed to high-dose anthracyclines and who had elevated NT-proBNP at baseline. Among 146 participants with baseline and follow-up NT-proBNP levels, 32 had elevated NT-proBNP and were included in this sub-analysis. At two years, carvedilol significantly improved LVWT/Dz (+ 1.5 in the carvedilol arm, *P* = 0.007) and reduced LVESWS (-17.15, *P* = 0.011), left ventricular end-diastolic diameter (LVEDD) (-0.26, *P* = 0.032), left ventricular end-systolic diameter (LVESD) (-0.31, *P* = 0.002), left ventricular end-diastolic volume (LVEDV) (-23.82, *P* = 0.002), and left ventricular end-systolic volume (LVESV) (-12.26, *P* = 0.008). Participants in this subgroup were older (34.0 ± 9.8 years) and had higher anthracycline exposure (415 ± 120 mg/m²) compared to the parent trial. The study concluded that carvedilol may benefit high-risk survivors with elevated NT-proBNP, but further trials are needed to confirm its role in preventing heart failure [19].

Agarwal et al. [17] investigated the therapeutic role of carvedilol in pediatric oncology patients with anthracycline-induced cardiomyopathy. In this cohort study, 34 patients with established anthracycline-induced cardiomyopathy were evaluated. Twenty-one patients received carvedilol, initiated at low doses (3.25 mg twice daily or 0.025 mg/kg per dose twice daily) and titrated to the maximally tolerated dose, while 13 patients did not receive beta-blocker therapy and served as a comparator group. Patients treated with carvedilol demonstrated statistically significant improvements in echocardiographic and clinical parameters. Mean LVEF increased from 32.2% to 47.7% (*P* < 0.01) and FS from 17.2% to 25.4% (*P* < 0.01) in carvedilol group. Furthermore, NYHA functional class improved from 3.24 to 1.24 (*P* < 0.01). In contrast, patients who did not receive carvedilol exhibited no significant changes in ventricular function or symptomatic status. Carvedilol was well tolerated in all treated patients. Notably, one patient in the non-carvedilol group died of progressive heart failure and another required cardiac transplantation. Thereby, carvedilol was safe and confers significant improvements in both ventricular function and clinical outcomes in pediatric patients with anthracycline-induced cardiomyopathy [17].

Pozza et al. [27] conducted a retrospective study which included 66 consecutive pediatric patients with ALL. The mean age was 9.4 ± 5.2 years. All patients received anthracycline-containing chemotherapy. Echocardiographic monitoring was performed at baseline, before each anthracycline cycle, and at the end of therapy. Pathological cardiac dysfunction was defined as an absolute GLS value worse than − 19%, a > 10% decline in EF, or an absolute EF below 53%. Among the 66 patients, 36 developed pathological GLS values during treatment. Of these, 24 patients were promptly treated with carvedilol from the time the abnormal GLS was detected until the end of therapy. Twelve patients with abnormal GLS did not receive carvedilol, mainly because their abnormal strain was detected retrospectively through off-line analysis rather than in real time. In the carvedilol group, GLS initially worsened during treatment (from − 20.5 ± 2.4 at baseline to -17.2 ± 1.3 at nadir), but recovered to baseline by the end of chemotherapy (-20.0 ± 1.7, P = NS for baseline vs. end), indicating preserved cardiac function. In contrast, patients not receiving carvedilol showed persistent impairment: GLS worsened from − 20.7 ± 2.3 at baseline to -17.0 ± 1.8 at nadir, and remained significantly reduced at treatment completion (-18.4 ± 2.4, *P* < 0.001 vs. baseline). Thus, early treatment with carvedilol in response to subclinical dysfunction detected by GLS preserved cardiac function during anthracycline therapy [27].

Shaddy and colleagues [29] described a case series evaluating the efficacy and safety of metoprolol in three pediatric patients with doxorubicin-induced cardiomyopathy. The onset of congestive heart failure occurred between two and 13 years after chemotherapy. All patients exhibited severe LV dysfunction at baseline, with radionuclide LVEF values ranging from 14% to 30% and NYHA functional class III-IV symptoms, despite prior therapy with digoxin, diuretics, ACE inhibitors, or hydralazine. Metoprolol was initiated at 6.25 mg twice daily and up-titrated to individualized maximum tolerated doses (75–125 mg/day). Treatment duration ranged from 0.5 to 2.5 years. Serial echocardiographic and radionuclide assessments showed that LVEF increased from 14 to 30% at baseline to 38–57% after therapy, and FS improved from 8 to 10% to 21–30%. Clinical status improved in parallel, with NYHA functional class reduced from III-IV to I-II. Two patients experienced such significant recovery in cardiac function and symptoms, while the third patient, who had also received thoracic irradiation, derived only partial benefit and subsequently underwent heart transplantation four years later. Consequently, beta-adrenergic blockade with metoprolol may be both safe and effective in the management of pediatric anthracycline-induced cardiomyopathy [29].

#### Quality assessment results

The quality assessment of the included studies demonstrates generally robust methodology for clinical trials and moderate-to-variable rigor for observational designs. Among nine randomized trials evaluated with RoB 2, four trials [18, 19, 21, 30] were judged at low risk across all domains, whereas the study by El-Shitany et al. exhibited some concerns [20], and four trials [16, 17, 26, 28] showed overall high-risk judgments mainly due to lack of detailed information **(Table S2)**. Cohort studies scored modestly on the NOS checklist. The studies by Lipshultz et al. and Harrington et al. [22, 24] each scored 4–5 out of 9 stars, while Pozza et al. [27] achieved 6 stars through stronger comparability and outcome ascertainment **(Table S3)**. Case-series assessments via the JBI tool revealed incomplete inclusion of participants and site/clinic descriptions, though clinical details and outcome reporting were generally satisfactory [23, 29] **(Table S4)**. Finally, both case reports met al.l JBI criteria [25, 31] **(Table S5)**.

## Discussion

Present systematic review integrates the current evidence regarding the role of renin-angiotensin-aldosterone system (RAAS) modulators (i.e., ACE inhibitors and ARBs) and beta-blockers in preventing or managing anthracycline-induced cardiotoxicity among pediatric cancer survivors. Across 16 studies, our findings emphasize consistent reports supporting the use of these medications to attenuate LV dysfunction, improve cardiac biomarkers, and, in some cases, reduce adverse clinical outcomes. According to the body of evidence, ACEi/ARBs and beta-blockers would be more effective in preventive strategies compared to reversal-oriented treatment approaches. However, the magnitude and durability of benefit remain unclear due to the methodological heterogeneity, limited sample sizes, and variations in study populations and interventions.

### Pathophysiologic rationale for ACEi/ARB and beta-blocker cardioprotection

Pediatric patients are more susceptible to anthracycline-induced cardiotoxicity than adult patients, which can be attributed to age-related variations in cardiac physiology and molecular pathways. The immature pediatric heart responds differently to anthracycline-induced oxidative stress and cell death mechanisms [32, 33]. Anthracyclines trigger cardiomyocyte injury through topoisomerase-IIβ-mediated DNA damage, reactive oxygen species, iron overload, and maladaptive autophagy, leading to eccentric remodeling and rising wall stress [1, 34]. Additional mechanisms, including ferroptosis and pyroptosis, contribute to cardiomyocyte damage. Finally, the development of interstitial fibrosis impairs heart architecture and function [33, 35].

The activation of neurohormones, especially the RAAS and sympathetic nervous system, exacerbates this physiological stress, providing a mechanistic rationale for using both ACEi/ARB and beta-blockers as therapeutic interventions for preventing secondary complications [34, 36, 37]. ACE inhibitors or ARBs primarily work by reducing angiotensin II formation or action and subsequent diminished aldosterone release, resulting in prevention of cardiac remodeling [38, 39]. In this case, ventricular remodeling prevention occurs mainly through inhibition of RAAS, which reduces angiotensin II-mediated vasoconstriction, myocardial contractility, and hypertrophy. On the other hand, reduction in afterload through vasodilation decreases ventricular wall stress, reducing myocardial oxygen demand and preventing pathological hypertrophy. Studies demonstrate that ACE inhibitors prevent matrix metalloproteinase (MMP) activity, particularly MMP-2, which is an important mechanism for preventing negative structural and functional changes in heart failure models [40]. The cardioprotective effect of beta-blockers, especially carvedilol, is mainly explained by beta-adrenergic blockade and potent antioxidant properties [41]. The antioxidant activity directly reduces catecholamine-driven oxygen radical production, providing short-term protection against anthracycline-induced damage [42, 43].

According to the body of evidence, ACEi/ARBs and beta-blockers are more effective in preventive strategies compared to reversal-oriented treatment approaches. Studies comparing different timings of ACE inhibitor initiation demonstrate that early intervention during the remodeling process yields superior results as compared to treatment initiated after established dysfunction [20, 44, 45]. Continued DNA damage and progressive myocyte loss that are initiated following anthracycline cardiotoxicity take place despite ongoing RAAS blockade. Similarly, while beta-blockers prevent acute hemodynamic stress and reduce oxidative burden, they cannot reverse established myocyte loss or prevent the progressive DNA damage cascade in late survivors. Thereby, the inability of neurohormonal therapies to prevent topoisomerase-IIβ-mediated DNA damage explains why cardioprotection diminishes over time [38, 46].

### Interpretation of the results

#### ACE inhibitors and ARBs

Many studies included in the present systematic review evaluated ACE inhibitors, particularly enalapril and captopril, either as prophylactic or therapeutic interventions. Clinical trials demonstrated that enalapril therapy mitigates early declines in LVEF and attenuates rises in biomarkers such as NT-proBNP and cTnI in children receiving anthracyclines, supporting its role in cardioprotection. While some LV function parameters improved with ACE inhibitors among RCTs, no benefits in exercise capacity was noted, raising questions about the clinical relevance of surrogate outcomes. Observational studies further highlighted improvements in echocardiographic indices, including FS, strain parameters, and LV mass when ACE inhibitors were initiated after anthracycline-induced cardiotoxicity had developed. Nevertheless, the evidence on long-term efficacy remains mixed. For instance, Lipshultz et al. reported initial improvement in LV dimensions and function with enalapril, which later waned over extended follow-up of 10 years, underscoring the challenge of sustained cardioprotection. Similarly, Harrington et al. showed GLS improvements in the first year of ACEi/ARB therapy which remained stable in the following years without further change. As a result, while some childhood survivors maintained improved cardiac function, others experienced progressive deterioration despite continued therapy, indicating that ACE inhibition may delay but not fully prevent late cardiomyopathy. Furthermore, it was established that the higher blood pressure, particularly diastolic blood pressure at initiation of ACE inhibitors was a strong predictor of better LV-related outcomes during treatment. Besides, side effects such as hypotension and fatigue were more prevalent in ACE inhibitor arms of RCTs, underlining the need to balance potential benefits with tolerability.

Recent evidence on the use of ARBs and ARNIs in pediatric settings are limited but encouraging. Notably, a case report of valsartan/sacubitril demonstrated rapid reversal of decompensated heart failure in a child refractory to conventional therapies. Although anecdotal, this highlights the potential of ARNIs as an emerging therapeutic option in high-risk populations. Overall, these findings support the use of ACE inhibitors and related agents, particularly when initiated early or in patients with established LV dysfunction, though long-term durability remains unclear.

Our results support the concept that inhibition of angiotensin-converting-enzyme promotes favorable remodeling of the afterload-stressed ventricle but, at pediatric doses administered, it may not reverse established systolic dysfunction [41]. This mechanism-specific efficacy can be reduced by frequent incidence of hypotensive symptoms, that may compromise adherence [30, 42]. As it was noted, enalapril with a maximal dose of 0.15 mg/kg/day resulted in a documentation of 15 cases of hypertension among 69 pediatric cancer survivors, underscoring the limitation in up-titrating ACE inhibitors to optimal doses in children [30].

Comparing with adult trials, the 2024 PROACT study revealed no difference in troponin level, GLS, or LVEF after two years between enalapril and standard care groups among breast cancer patients who had been on high-dose of doxorubicin [43]. However, Gupta et al. demonstrated promising results regarding the protective role of enalapril among pediatric cancer patients. The difference could be potentially justified by probable higher rate of preexisting comorbidities in adults, the higher cumulative doses of doxorubicin (i.e., ≥ 300 mg/m² in adults vs. ≥200 mg/m² in children), and reduced cardiac plasticity and capacity for remodeling in older patients, which all may overwhelm any protective effect of enalapril. Additionally, systematic review and meta-analyses of adult cancer patients showed modest therapeutic effect of ACEi/ARBs toward improving LVEF during at-least six months of follow-up [44, 47].

#### Beta-blockers

Evidence for beta-blockers, particularly carvedilol, has grown in recent years. Several randomized and observational studies demonstrated significant improvements in ventricular function, strain imaging, and NYHA class with carvedilol use among children with anthracycline-induced cardiomyopathy. Importantly, carvedilol was well tolerated, with only minor adverse events reported. Notably, carvedilol prevented persistent strain abnormalities and troponin elevation when initiated concurrently with anthracyclines administration. Larger RCTs, such as the PREVENT-HF trial, did not demonstrate a significant effect on the primary outcome of cardiac remodeling, although post-hoc analyses suggested potential benefit in high-risk subgroups with elevated NT-proBNP. These findings imply that the cardioprotective effect of carvedilol may be most pronounced in survivors with early subclinical or biochemical evidence of dysfunction rather than in all exposed patients.

Limited data exist on other beta-blockers. Case series of metoprolol have reported partial to full recovery of LVEF in children with severe cardiomyopathy, suggesting that beta-blockade may restore function even in advanced disease. Collectively, the body of evidence indicates that beta-blockers, either alone or in combination with ACE inhibitors, can play a crucial role in mitigating both subclinical and overt cardiotoxicity.

The short-term benefits of beta-blockers align with multiple meta-analyses conducted in adult trials [10, 38, 39, 45, 46]. A network meta-analysis encompassing 1,977 adult patients with breast cancer showed that after trimetazidine monotherapy or its combination with dexrazoxane, the combination of an ACEi/ARB and a beta-blocker ranked third intervention for LVEF enhancement compared to the control, according to the Surface Under the Cumulative Ranking (SUCRA) evaluation of ranking probabilities (MD: 5.97, SUCRA: 78.3%) [10].

Furthermore, a comprehensive 2018 meta-analysis of eight randomized controlled trials (633 patients) demonstrated that prophylactic carvedilol significantly reduced rates of low LVEF favoring the carvedilol group (3.2% vs. 5.8%) and provided smaller reductions in LVEF compared to placebo (mean differences [MD]: 2.41) [38]. Similarly, a 2019 meta-analysis of 11 trials (940 participants) confirmed that beta-blockers were associated with a 71% reduction in symptomatic heart failure risk (risk ratio [RR]: 0.29, 95% CI: 0.10 to 0.85) and improved s’ (MD: 0.78) in parallel with reduced LVESD (MD: -3.19) and LVEDD (MD: -2.28). Beta-blockers also improved strain and strain rate when compared with placebo. Moreover, beta-blockers reduced the risk of cTnI elevation > 0.04 ng/ml (RR: 0.60, 95% CI: 0.42 to 0.85) [45]. Although some significant associations were found toward favorable impact of beta-blockers for anthracycline-induced cardiotoxity among systematic reviews, it has been reported that the certainty of evidence for making a clinical decision is mainly low [39].

Besides, a 2020 systematic review revealed significant subgroup differences based on follow-up duration, with adult cancer patients receiving six months of beta-blockers therapy showed improved LVEF (MD = 6.48) in contrast to patients who received beta-blockers for less than six months (MD = − 0.05) [46]. It would be important to note that the majority of included studies in these systematic reviews had a short follow-up duration and the benefits of beta-blocker therapy may be waned in later years. The short-term benefits likely came from carvedilol’s combined beta-adrenergic blockade and antioxidant properties that reduce catecholamine-driven oxygen-radical production. In contrast, the absence of structural change over long follow-up periods which was evident in PREVENT-HF trial suggests that beta-blockade alone cannot overcome myocyte loss in late survivors.

### Implications for clinical practice

#### Risk stratification

Key determinants of increased risk of cardiotoxicity among childhood cancer survivors receiving anthracyclines include demographic factors such as younger ages at exposure and female sex, genetic predispositions, cancer type, and cumulative dose of anthracyclines [48, 49]. Furthermore, children with trisomy 21 and those of Black ethnicity exhibit heightened susceptibility [50]. Bloodstream infections, including sepsis, have been identified as a major contributor to the development and worsening of cardiotoxicity among children exposed to anthracyclines, with sepsis-associated cardiac dysfunction being closely linked to mortality [5].

Genetic polymorphisms significantly influence individual susceptibility to anthracycline cardiotoxicity. Genetic variations such as SLC28A3, UGT1A6, and RARG have been identified as risk modifiers. Specifically, the SLC28A3 protective variant benefits more at higher anthracycline dosages, while UGT1A6 and RARG risk variants show greater effect at lower levels [51, 52]. Integrating genetic screening into pediatric oncology protocols could enable proactive identification of high-risk individuals and personalize prevention strategies [53, 54].

Treatment-related factors, including anthracycline dosage and concurrent radiotherapy, further modulate risk profiles. The relationship between cumulative anthracycline dose and cardiotoxicity is well established, though no entirely “safe” threshold dose has been identified [50]. Studies show that doses exceeding 200–300 mg/m² substantially increase the risk of cardiotoxicity [24], but older landmark cohorts have recognized a 550 mg/m² threshold conferring significant cardiotoxic risk [55]. However, cardiotoxicity can manifest even at lower doses, influenced by individual susceptibility and concomitant factors.

Recently, studies identified scores based on baseline troponin, strain imaging, and polygenic risk scores to improve the identification of survivors most likely to benefit from neurohormonal inhibition [56, 57]. Also, novel risk stratification incorporates genetic variants influencing drug metabolism, transport, and cardiomyocyte susceptibility, facilitating the identification of high-risk individuals who may benefit from intensified monitoring or prophylactic interventions [57].

#### Continuous monitoring

Serial echocardiography and checking cardiac biomarkers could guide titration and discontinuation decisions [58]. While echocardiography remains a standard modality for cardiotoxicity surveillance, LVEF lacks sensitivity for detecting subclinical or early myocardial dysfunction, often only declining after significant myocardial injury [3]. Also, the timing of echocardiographic evaluations varies, but early post-treatment monitoring and long-term follow-up are important given the progressive nature of anthracycline cardiotoxicity [1, 8]. Advanced echocardiographic techniques such as speckle tracking echocardiography (STE) can provide more sensitivity in detecting subtle myocardial deformation abnormalities, preceding overt cardiac dysfunction. Evidence supports the predictive value of combining STE-derived metrics in identifying at-risk pediatric patients undergoing anthracycline therapy [59, 60].

Additionally, the utilization of cardiac biomarkers such as cardiac troponins (cTnI, high-sensitivity cTnT) and natriuretic peptides (BNP, pro-BNP) complements imaging-based monitoring by providing biochemical evidence of myocardial injury and stress. Elevated troponin levels correlate with echocardiographic abnormalities and predict cardiotoxic events [59]. Biomarker elevation often precedes functional impairment detected by imaging, offering a window for early therapeutic intervention [61, 62].

#### Incorporating ACEi/ARBs and beta-blockers

The evidence suggests that ACEi/ARBs and beta-blockers, particularly when introduced early and guided by careful surveillance, may protect against anthracycline-induced cardiotoxicity in children. While the benefits appear more robust for surrogate endpoints such as LVEF, FS, and GLS, some studies also indicate improvements in EFS and reduced heart failure incidence. Importantly, these therapies were generally well tolerated, although ACE inhibitors may pose risks of hypotension and fatigue, particularly with long-term use. Given the growing population of childhood cancer survivors at risk of late-onset cardiomyopathy, these findings support integrating cardioprotective agents into survivorship care protocols, especially for patients with high cumulative anthracycline exposure or early signs of dysfunction.

### Research gaps and future research priorities

Clinically, early initiation of cardioprotection strategies ACEi/ARBs and beta-blockers in high-risk children may delay cardiac functional decline. Still, the modest absolute effect sizes, small sample frames, and tolerability concerns challenge routine prophylaxis. Future trials should employ strain imaging and biomarker-guided enrichment to identify subgroups with residual adaptive myocardium. These potential cancer survivors can be recruited for exploring the efficacy and safety of combination of RAAS inhibitors and beta-blocker strategies in attenuation of anthracycline-induced cardiotoxicity. Extending surveillance for several years would be necessary to find out whether the observed cardioprotection benefits observed in children clinical trials translate into meaningful prevention of heart-failure events in adulthood. Future approaches may require targeting multiple pathways simultaneously, including topoisomerase-IIβ inhibition, iron chelation, ferroptosis inhibition, and neurohormonal blockade [63–65].

### Limitations

The included studies into present systematic review span diverse populations, cancers, and chemotherapy regimens, offering a broad view of cardioprotective strategies. However, limitations include the predominance of small single-center studies with underpowered clinical endpoints, inconsistent follow-up durations, variations in imaging modalities, and variable dosing regimens. The heterogeneity precluded meta-analysis and limits the ability to draw definitive conclusions regarding optimal drug choice, dose, or duration. Another limitation is the reliance on lower levels of evidence, including case reports, case series, and conference abstracts. While these sources provide less methodological rigor compared to randomized trials or large observational studies, they nevertheless offer valuable insights in pediatric cardio-oncology, where high-quality data remain scarce. Case-based evidence often illustrates unique clinical scenarios or the potential benefits of less commonly used agents, and conference abstracts may contain the most up-to-date findings not yet available in full publications. To balance their inclusion, we explicitly identified these studies, acknowledged their inherent limitations, and interpreted their findings with appropriate caution.

### Conclusion

ACEi/ARBs and beta-blockers continue to offer a theoretically reasonable, albeit clinically uncertain, strategy for preventing or managing anthracycline-related cardiotoxicity. While consistent benefits have been demonstrated for surrogate cardiac outcomes, the durability of protection and impact on long-term clinical endpoints remain unclear. Early initiation, vigilant monitoring, and combination therapy may enhance cardioprotection, however, high-quality clinical trials are required in order to establish definitive recommendations.

## Supplementary Information

Supplementary Material 1.

## Data Availability

The datasets analysed during the current study are available upon request from corresponding author.
