# Supplementary material for: Cardioprotective strategies of ACEi/ARBs and beta-blockers against anthracycline-induced cardiotoxicity in pediatric cancer survivors: a systematic review
Source: Cardiooncology. 2026 Feb 18;12:41. doi: 10.1186/s40959-026-00447-5 (PMC13020010; doi:10.1186/s40959-026-00447-5)
Supplement: Supplementary file 1 — Supplementary Material 1. [file 40959_2026_447_MOESM1_ESM.docx]

**Cardioprotective Strategies of ACEi/ARBs and Beta-Blockers Against Anthracycline-Induced Cardiotoxicity in Pediatric Cancer Survivors: A Systematic Review**

**Table S1.** Search strategy for PubMed, Scopus, and Web of Science databases.

| **Database**  **(search date)** | **step** | **Search strategy** | **Number of results** |
| --- | --- | --- | --- |
| PubMed  (8.16.2025) | #1 | "Adrenergic beta-Antagonists"[Mesh]  OR "beta antagonist*"[tiab] OR "beta-antagonist*"[tiab]  OR "beta blocker*"[tiab] OR "beta-blocker*"[tiab]  OR "β blocker*"[tiab] OR "β-blocker*"[tiab]  OR "beta adrenergic antagonist*"[tiab] OR "beta-adrenergic antagonist*"[tiab]  OR "beta adrenergic blocker*"[tiab] OR "beta-adrenergic blocker*"[tiab]  OR "beta adrenergic receptor blocker*"[tiab] OR "beta-adrenergic receptor blocker*"[tiab]  OR "beta blockade*"[tiab] OR "beta-blockade*"[tiab]  OR "β blockade*"[tiab] OR "β-blockade*"[tiab]  OR carvedilol[tiab] OR metoprolol[tiab] OR propranolol[tiab] OR nadolol[tiab]  OR timolol[tiab] OR sotalol[tiab] OR atenolol[tiab] OR bisoprolol[tiab]  OR esmolol[tiab] OR labetalol[tiab] OR nebivolol[tiab] OR carteolol[tiab] OR pindolol[tiab]  OR "Angiotensin-Converting Enzyme Inhibitors"[Mesh]  OR "angiotensin-converting enzyme inhibitor*"[tiab] OR "angiotensin converting enzyme inhibitor*"[tiab]  OR "angiotensin-converting enzyme antagonist*"[tiab] OR "angiotensin converting enzyme antagonist*"[tiab]  OR "ACE inhibitor*"[tiab] OR "ACE-inhibitor*"[tiab]  OR lisinopril[tiab] OR enalapril[tiab] OR ramipril[tiab] OR captopril[tiab]  OR perindopril[tiab] OR benazepril[tiab] OR quinapril[tiab] OR fosinopril[tiab]  OR trandolapril[tiab] OR moexipril[tiab] OR cilazapril[tiab]  OR "Angiotensin Receptor Antagonists"[Mesh]  OR "angiotensin receptor antagonist*"[tiab] OR "angiotensin-receptor antagonist*"[tiab]  OR "angiotensin II receptor antagonist*"[tiab] OR "angiotensin-II receptor antagonist*"[tiab]  OR "angiotensin II-receptor antagonist*"[tiab] OR "angiotensin-II-receptor antagonist*"[tiab]  OR "angiotensin 2 receptor antagonist*"[tiab] OR "angiotensin-2 receptor antagonist*"[tiab]  OR "angiotensin 2-receptor antagonist*"[tiab] OR "angiotensin-2-receptor antagonist*"[tiab]  OR "angiotensin receptor blocker*"[tiab] OR "angiotensin-receptor blocker*"[tiab]  OR "angiotensin II receptor blocker*"[tiab] OR "angiotensin-II receptor blocker*"[tiab]  OR "angiotensin II-receptor blocker*"[tiab] OR "angiotensin-II-receptor blocker*"[tiab]  OR "angiotensin 2 receptor blocker*"[tiab] OR "angiotensin-2 receptor blocker*"[tiab]  OR "angiotensin 2-receptor blocker*"[tiab] OR "angiotensin-2-receptor blocker*"[tiab]  OR losartan[tiab] OR valsartan[tiab] OR candesartan[tiab] OR irbesartan[tiab]  OR telmisartan[tiab] OR olmesartan[tiab] OR eprosartan[tiab] OR azilsartan[tiab] OR "ACEI/ARB*"[tiab] | 188,985 |
|  | #2 | "Child"[Mesh] OR "child*"[tiab] OR "Pediatrics"[Mesh] OR "pediatric*"[tiab] OR "paediatric*"[tiab] | 3,050,343 |
|  | #3 | "Neoplasms"[Mesh] OR “neoplasm*”[tiab] OR “tumor*”[tiab] OR "neoplasia*"[tiab] OR "cancer*"[tiab] OR "malignanc*"[tiab] OR "leukemia*"[tiab] OR "leukaemia*"[tiab] OR "lymphoma*"[tiab] OR "oncology"[tiab] | 5,513,941 |
|  | #4 | "hemangioma*"[tiab] OR "haemangioma*"[tiab] | 28,047 |
|  | #5 | #1 AND #2 AND #3 NOT #4 | 373 |
| Scopus  (8.16.2025) | #1 | TITLE-ABS-KEY("beta antagonist*" OR "beta-antagonist*"  OR "beta blocker*" OR "beta-blocker*"  OR "β blocker*" OR "β-blocker*"  OR "beta adrenergic antagonist*" OR "beta-adrenergic antagonist*"  OR "beta adrenergic blocker*" OR "beta-adrenergic blocker*"  OR "beta adrenergic receptor blocker*" OR "beta-adrenergic receptor blocker*"  OR "beta blockade*" OR "beta-blockade*"  OR "β blockade*" OR "β-blockade*"  OR carvedilol OR metoprolol OR propranolol OR nadolol  OR timolol OR sotalol OR atenolol OR bisoprolol  OR esmolol OR labetalol OR nebivolol OR carteolol OR pindolol  OR "angiotensin-converting enzyme inhibitor*" OR "angiotensin converting enzyme inhibitor*"  OR "angiotensin-converting enzyme antagonist*" OR "angiotensin converting enzyme antagonist*"  OR "ACE inhibitor*" OR "ACE-inhibitor*"  OR lisinopril OR enalapril OR ramipril OR captopril  OR perindopril OR benazepril OR quinapril OR fosinopril  OR trandolapril OR moexipril OR cilazapril  OR "angiotensin receptor antagonist*" OR "angiotensin-receptor antagonist*"  OR "angiotensin II receptor antagonist*" OR "angiotensin-II receptor antagonist*"  OR "angiotensin II-receptor antagonist*" OR "angiotensin-II-receptor antagonist*"  OR "angiotensin 2 receptor antagonist*" OR "angiotensin-2 receptor antagonist*"  OR "angiotensin 2-receptor antagonist*" OR "angiotensin-2-receptor antagonist*"  OR "angiotensin receptor blocker*" OR "angiotensin-receptor blocker*"  OR "angiotensin II receptor blocker*" OR "angiotensin-II receptor blocker*"  OR "angiotensin II-receptor blocker*" OR "angiotensin-II-receptor blocker*"  OR "angiotensin 2 receptor blocker*" OR "angiotensin-2 receptor blocker*"  OR "angiotensin 2-receptor blocker*" OR "angiotensin-2-receptor blocker*"  OR losartan OR valsartan OR candesartan OR irbesartan  OR telmisartan OR olmesartan OR eprosartan OR azilsartan OR "ACEI*ARB*") | 424,351 |
|  | #2 | TITLE-ABS-KEY("child*" OR "pediatric*" OR "paediatric*") | 4,368,344 |
|  | #3 | TITLE-ABS-KEY("neoplasm*" OR "tumor*" OR "neoplasia*" OR "cancer*" OR "malignanc*" OR "leukemia*" OR "leukaemia*" OR "lymphoma*" OR "oncology") | 7,020,403 |
|  | #4 | TITLE-ABS-KEY("hemangioma*" OR "haemangioma*") | 62,228 |
|  | #5 | #1 AND #2 AND #3 AND NOT #4 | 2,047 |
| Web of science  (8.16.2025) | #1 | TS=("beta antagonist*" OR "beta-antagonist*"  OR "beta blocker*" OR "beta-blocker*"  OR "β blocker*" OR "β-blocker*"  OR "beta adrenergic antagonist*" OR "beta-adrenergic antagonist*"  OR "beta adrenergic blocker*" OR "beta-adrenergic blocker*"  OR "beta adrenergic receptor blocker*" OR "beta-adrenergic receptor blocker*"  OR "beta blockade*" OR "beta-blockade*"  OR "β blockade*" OR "β-blockade*"  OR carvedilol OR metoprolol OR propranolol OR nadolol  OR timolol OR sotalol OR atenolol OR bisoprolol  OR esmolol OR labetalol OR nebivolol OR carteolol OR pindolol  OR "angiotensin-converting enzyme inhibitor*" OR "angiotensin converting enzyme inhibitor*"  OR "angiotensin-converting enzyme antagonist*" OR "angiotensin converting enzyme antagonist*"  OR "ACE inhibitor*" OR "ACE-inhibitor*"  OR lisinopril OR enalapril OR ramipril OR captopril  OR perindopril OR benazepril OR quinapril OR fosinopril  OR trandolapril OR moexipril OR cilazapril  OR "angiotensin receptor antagonist*" OR "angiotensin-receptor antagonist*"  OR "angiotensin II receptor antagonist*" OR "angiotensin-II receptor antagonist*"  OR "angiotensin II-receptor antagonist*" OR "angiotensin-II-receptor antagonist*"  OR "angiotensin 2 receptor antagonist*" OR "angiotensin-2 receptor antagonist*"  OR "angiotensin 2-receptor antagonist*" OR "angiotensin-2-receptor antagonist*"  OR "angiotensin receptor blocker*" OR "angiotensin-receptor blocker*"  OR "angiotensin II receptor blocker*" OR "angiotensin-II receptor blocker*"  OR "angiotensin II-receptor blocker*" OR "angiotensin-II-receptor blocker*"  OR "angiotensin 2 receptor blocker*" OR "angiotensin-2 receptor blocker*"  OR "angiotensin 2-receptor blocker*" OR "angiotensin-2-receptor blocker*"  OR losartan OR valsartan OR candesartan OR irbesartan  OR telmisartan OR olmesartan OR eprosartan OR azilsartan OR "ACEI/ARB*") | 202,377 |
|  | #2 | TS=("child*" OR "pediatric*" OR "paediatric*") | 3,010,097 |
|  | #3 | TS=("neoplasm*" OR "tumor*" OR "neoplasia*" OR "cancer*" OR "malignanc*" OR "leukemia*" OR "leukaemia*" OR "lymphoma*" OR "oncology") | 5,602,707 |
|  | #4 | TS=("hemangioma*" OR "haemangioma*") | 28,396 |
|  | #5 | #1 AND #2 AND #3 NOT #4 | 306 |

**Table S2.** Quality assessment of included clinical trials using risk-of-bias tool for randomized trials (RoB 2).

| **Study (Year)** | **1. Randomization Process** | **2. Deviations from Intended Interventions** | **3. Missing Outcome Data** | **4. Measurement of the Outcome** | **5. Selection of Reported Result** | **Overall RoB 2 Judgment** |
| --- | --- | --- | --- | --- | --- | --- |
| Armenian et al. (2024) (1) | Low risk | Low risk | Low risk | Low risk | Low risk | Low risk |
| Armenian et al. (2024) post-hoc analysis (2) | Low risk | Low risk | Low risk | Low risk | Low risk | Low risk |
| El-Shitany et al. (2012) (3) | Some concerns | Some concerns | Low risk | Some concerns | Low risk | Some concerns |
| Gupta et al. (2018) (4) | Low risk | Low risk | Low risk | Low risk | Low risk | Low risk |
| Silber et al. (2004) (5) | Low risk | Low risk | Low risk | Low risk | Low risk | Low risk |
| Agarwal et al. (2005) (6) | Some concerns | Some concerns | Some concerns | Low risk | High risk | High risk |
| Mandric et al. (2008) (7) | Some concerns | Some concerns | Some concerns | Low risk | High risk | High risk |
| Selim et al. (2025) (8) | Some concerns | Some concerns | Some concerns | Low risk | High risk | High risk |
| Seth et al. (2023) (9) | Some concerns | Some concerns | Some concerns | Low risk | High risk | High risk |

| **Study (Year)** | **Selection (0-4★)** | **Comparability (0-2★)** | **Outcome (0-3★)** | **Total Score** |
| --- | --- | --- | --- | --- |
| Lipshultz et al. (2002) (10) | 2/4 | 0/2 | 2/3 | 4/9 |
| Harrington et al. (2018) (11) | 2/4 | 0/2 | 3/3 | 5/9 |
| Pozza et al. (2025) (12) | 2/4 | 1/2 | 3/3 | 6/9 |

**Table S3.** Quality assessment of included cohort studies using Newcastle Ottawa Scale (NOS) checklist.

| **Study (Year)** | **1. Clear inclusion criteria** | **2. Standard measurement of condition** | **3. Valid identification methods** | **4. Consecutive inclusion** | **5. Complete inclusion** | **6. Demographics reported** | **7. Clinical info reported** | **8. Outcomes reported** | **9. Site/clinic characteristics** | **10. Statistical analysis** |
| --- | --- | --- | --- | --- | --- | --- | --- | --- | --- | --- |
| Hauser et al. (2000) (13) | Yes | Yes | Yes | Unclear | No | Yes | Yes | Yes | No | N/A |
| Shaddy et al. (1995) (14) | No | Yes | Yes | No | No | Yes | Yes | Yes | No | N/A |

**Table S4.** Quality assessment of included case-series using Joanna Briggs Institute (JBI) checklist.

| **Study (Year)** | **1. Patient details** | **2. Clear history** | **3. Current clinical condition** | **4.**  **Diagnostic tests** | **5. Intervention described** | **6. Post‐intervention condition** | **7. Adverse events** | **8. Key lessons** |
| --- | --- | --- | --- | --- | --- | --- | --- | --- |
| Lo et al. (2021) (15) | Yes | Yes | Yes | Yes | Yes | Yes | Yes | Yes |
| Tony et al. (2021) (16) | Yes | Yes | Yes | Yes | Yes | Yes | Yes | Yes |

**Table S5.** Quality assessment of included case-reports using Joanna Briggs Institute (JBI) checklist**.**

**References**

1. Armenian SH, Hudson MM, Lindenfeld L, Chen S, Chow EJ, Colan S, et al. Effect of carvedilol versus placebo on cardiac function in anthracycline-exposed survivors of childhood cancer (PREVENT-HF): a randomised, controlled, phase 2b trial. Lancet Oncol. 2024;25(2):235-45.

2. Armenian SH, Hudson MM, Lindenfeld L, Chen S, Chow EJ, Colan S, et al. Carvedilol to Improve Cardiac Remodeling in Anthracycline-Exposed Childhood Cancer Survivors: Subgroup Analysis of COG ALTE1621. JACC: CardioOncology. 2024;6(5):791-3.

3. El-Shitany NA, Tolba OA, El-Shanshory MR, El-Hawary EE. Protective Effect of Carvedilol on Adriamycin-Induced Left Ventricular Dysfunction in Children With Acute Lymphoblastic Leukemia. Journal of Cardiac Failure. 2012;18(8):607-13.

4. Gupta V, Kumar Singh S, Agrawal V, Bali Singh T. Role of ACE inhibitors in anthracycline-induced cardiotoxicity: A randomized, double-blind, placebo-controlled trial. Pediatr Blood Cancer. 2018;65(11):e27308.

5. Silber JH, Cnaan A, Clark BJ, Paridon SM, Chin AJ, Rychik J, et al. Enalapril to prevent cardiac function decline in long-term survivors of pediatric cancer exposed to anthracyclines. J Clin Oncol. 2004;22(5):820-8.

6. Agarwal R, Tong A, Yusuf SW, Wells R, Jaffe N, Burrer N, et al. Carvedilol for anthracylcine-induced heart failure in pediatric oncology patients. Journal of Cardiac Failure. 2005;11(6):S188-S.

7. Mandric C, Dimitriu AG, Miron I, Dimitriu L. The utility of enalapril treatement in anthracyclines-induced cardiac injury in children with malignancies-preliminary study. European Heart Journal. 2008;29:434-.

8. Selim Z, Gouda H, Albeltagi D, Mosaad M, Abdelmassih A, Kamal M, et al. Revisiting the role of angiotensin-converting enzyme inhibitors in preventing chemotherapy cancer related cardiac disease in paediatric malignant bone tumours patients. European Heart Journal Supplements. 2025;27(Supplement_6):suaf083. 06.

9. Seth R, Seth S, Bansal S, Setlur K, Gupta AK, Meena JP, et al. A STUDY OF ROLE OF CARDIO PROTECTION (ACEI PLUS BETA BLOCKADE) IN EARLY CHEMOTHERAPY INDUCED CARDIOTOXICITY IN PEDIATRIC LEUKEMIA DURING CHEMOTHERAPY. Pediatric Blood & Cancer. 2023;70.

10. Lipshultz SE, Lipsitz SR, Sallan SE, Simbre VC, Shaikh SL, Mone SM, et al. Long-term enalapril therapy for left ventricular dysfunction in doxorubicin-treated survivors of childhood cancer. Journal of Clinical Oncology. 2002;20(23):4517-22.

11. Harrington JK, Richmond ME, Fein AW, Kobsa S, Satwani P, Shah A. Two-Dimensional Speckle Tracking Echocardiography-Derived Strain Measurements in Survivors of Childhood Cancer on Angiotensin Converting Enzyme Inhibition or Receptor Blockade. Pediatric Cardiology. 2018;39(7):1404-12.

12. Pozza A, Somigli C, Putti MC, Biffi A, Di Salvo G, Castaldi B. Early detection of cardiotoxicity in paediatric acute lymphoblastic leukaemia: the use of carvedilol in a single centre experience. European Heart Journal Supplements. 2025;27.

13. Hauser M, Wilson N. Anthracycline induced cardiomyopathy: Successful treatment with angiotensin converting enzyme inhibitors. European Journal of Pediatrics. 2000;159(5):389.

14. Shaddy RE, Olsen SL, Bristow MR, Taylor DO, Bullock EA, Tani LY, et al. Efficacy and safety of metoprolol in the treatment of doxorubicin-induced cardiomyopathy in pediatric patients. American Heart Journal. 1995;129(1):197-9.

15. Lo SH, Liu YC, Dai ZK, Chen IC, Wu YH, Hsu JH. Case Report: Low Dose of Valsartan/Sacubitril Leads to Successful Reversal of Acute Heart Failure in Chemotherapy-Induced Cardiomyopathy. Frontiers in Pediatrics. 2021;9.

16. Tony S, Mevada R, Joshi N. Anthracycline-related acute cardiotoxicity in a very young Omani patient with acute myeloid leukaemia. Cardiology in the Young. 2021;31(3):464-7.
